# 

**Published:** 1945

**Authors:** 


### CONTENTS.

| | Page |
|---|---|
| **Gastroscopy: A Survey.** By Norman C. Tanner, M.B., Ch.B., F.R.C.S. .. | 1 |
| **Acute Inversion of the Uterus Following Delivery.** By Coralie Rendle-Short, M.B., Ch.M., M.R.C.O.G. .. | 9 |
| **Observations on Some Rock Carvings of Surgical Instruments in East Africa.** By M. Nierenstein, D.Sc. .. | 14 |
| **Selection of Medical Students.** By Frank Bodman, M.D. .. | 18 |
| **Reviews of Books** .. | 22 |
| **Editorial Notes** .. | 27 |
| **Obituaries**: John Dacre, M.R.C.S., L.R.C.P. .. | 31 |
| Ernest William Hey Groves, M.S., F.R.C.S. .. | 31 |
| Cecil Augustus Joll, M.D., B.S., B.Sc., F.R.C.S. .. | 34 |
| Leonard Newsom Morris, M.B., Ch.B., F.R.C.S., M.R.C.S., L.R.C.P. .. | 35 |
| Norman Lloyd Price, M.D., M.R.C.P. .. | 35 |
| Charles Ferrier Walters, F.R.C.S. .. | 35 |
| David Charles Rayner, Ch.M., F.R.C.S. .. | 36 |
| **Meetings of Societies** .. | 37 |
| **The Medical Library of the University of Bristol** .. | 38 |
| **Publications Received** .. | 41 |
| **Local Medical Notes** .. | 42 |

